# A 6-Year Single Center Experience in Neonatal Aortic Arch Surgery with Whole-Body Perfusion: Showing the Perks by Strict Propensity Score Matching

**DOI:** 10.3390/jcm14030843

**Published:** 2025-01-27

**Authors:** Isabelle Doll, Rodrigo Sandoval Boburg, Rafal Berger, Christian Jörg Rustenbach, Walter Jost, Jörg Michel, Harry Magunia, Christian Schlensak

**Affiliations:** 1 Department of Thoracic and Cardiovascular Surgery, University Hospital Tübingen, Eberhard-Karls-University Tübingen, Hoppe-Seyler-Str. 3, 72076 Tübingen, Germany; rodrigo.sandoval-boburg@med.uni-tuebingen.de (R.S.B.); rafal.berger@med.uni-tuebingen.de (R.B.); christian.rustenbach@med.uni-tuebingen.de (C.J.R.); walter.jost@med.uni-tuebingen.de (W.J.); christian.schlensak@med.uni-tuebingen.de (C.S.); 2 Department of Pediatric Cardiology and Intensive Care Medicine, University Hospital Tübingen, Eberhard-Karls-University Tübingen, Hoppe-Seyler-Str. 3, 72076 Tübingen, Germany; joerg.michel@med.uni-tuebingen.de; 3 Department of Anesthesiology and Intensive Care Medicine, University Hospital Tübingen, Eberhard-Karls-University Tübingen, Hoppe-Seyler-Str. 3, 72076 Tübingen, Germany; harry.magunia@med.uni-tuebingen.de

**Keywords:** whole-body perfusion, lower body perfusion, antegrade cerebral perfusion, neonatal aortic arch surgery, mild hypothermia

## Abstract

**Objectives**: Perfusion strategy is crucial for the outcome of neonatal aortic arch surgery. This study investigates Whole-Body Perfusion to potentially improve postoperative outcomes for neonates, addressing a significant gap in current research. **Methods**: Retrospective analysis was conducted for neonates receiving aortic arch reconstruction in our institution: 33 patients were treated with Antegrade Cerebral Perfusion (ACP, 2014–2017) and 61 patients with Whole-Body Perfusion (WBP, 2017–2022). After strict Propensity Score Matching, 20 patients were analyzed in each group. WBP consists of ACP and Lower Body Perfusion (LBP), achieved through a femoral arterial sheath. **Results**: Patients with WBP had a shorter time on Cardiopulmonary Bypass (86.65 ± 25.47 vs. 172.95 ± 60.12 min) and Cross-Clamp time (46.70 ± 18.48 vs. 91.30 ± 40.10 min) (*p* ≤ 0.001). Lactate at the time of reperfusion and after 24 h was lower in the WBP group (1.73 ± 0.63 vs. 4.29 ± 1.61, *p* < 0.001; 1.45 ± 0.57 vs. 2.09 ± 0.96 mmol/L, *p* = 0.026). Patients with WBP needed significantly fewer intraoperative transfusions of Red Blood Cells, Fresh Frozen Plasma and Platelets (*p* ≤ 0.001). WBP patients had a shorter time on ventilator (5.15 ± 4.05 vs. 10.00 ± 8.72 days, *p* = 0.01) and a higher urine output after 24 h (200.85 ± 100.87 vs. 118.10 ± 82.33 mL, *p* = 0.002). **Conclusions**: Patients treated with WBP received significantly fewer intraoperative transfusions and had a shorter time on extracorporeal circulation and ventilator. Furthermore, there was a trend for reduced multiorgan dysfunction.

## 1. Introduction

Aortic arch surgery in neonates and infants is a challenging procedure for the whole surgical team. Initially performed under Deep Hypothermic Cardiac Arrest (DHCA), many severe intra- and postoperative complications result. This makes it a high-risk procedure [1,2]. It is well known that reduced visceral blood flow is linked with renal failure, which is accompanied by a higher mortality [3]. Later, the refined method of Antegrade Cerebral Perfusion (ACP) was introduced. By minimizing the time of cerebral circulatory arrest, especially neurological complications, could be reduced [4]. This is the standard method used in most centers nowadays [5]. Still, there is a significant period with hardly any perfusion of the abdominal organs, which is not addressed by this method. Therefore, experimental approaches are trying to achieve Whole-Body Perfusion (WBP) through surgery. This could be obtained by direct cannulation of the descending aorta, either through a lateral thoracotomy or a posterior pericardial incision [6]. Our institution was one of the first to pioneer in the development of a new and feasible technique for WBP. Since 2017, we have used an arterial sheath in the femoral artery through which the lower body and abdominal organs get perfused simultaneously (Lower Body Perfusion (LBP)). This method was first described by Rajagopal et al. [1]. Our early results showed that common complications, such as postoperative hepatic and renal failure, could be reduced significantly, as well as the need for blood transfusions [7,8,9]. Overall, there is only scarce data regarding applications and outcomes of WBP in a pediatric cohort.

We achieved good clinical experience with our method with which we treated numerous patients over the last few years. The aim of this study was to reinforce our results by strict statistical Propensity Score Matching and contribute to the rare existing data in this field.

## 2. Materials and Methods

### 2.1. Ethical Statement

Approval for this retrospective study was obtained from the local institutional review board and ethics committee of the University of Tübingen (Project number 461/2019BO2) on 10 February 2021. Due to the retrospective character of this study, written consent was waived.

### 2.2. Patient Selection

This study was conducted retrospectively at the University Hospital Tübingen, Germany. All pediatric patients aged one and under who underwent aortic arch surgery in 2014–2022 were included. Operative procedures were the Norwood procedure, Damus–Kaye–Stansel anastomosis or aortic arch reconstruction due to hypoplasia either as primary or redo surgery.

All surgeries were performed by the same team of surgeons, anesthesiologists and perfusionist. Before January 2017, all patients undergoing any of these procedures were treated with ACP and moderate hypothermia (<30 °C). After January 2017, a WBP technique was established at our center consisting of the standard ACP with LBP and mild hypothermia (30–32 °C). All pediatric patients undergoing aortic arch surgery after this date were treated with WBP.

**Figure 1:**
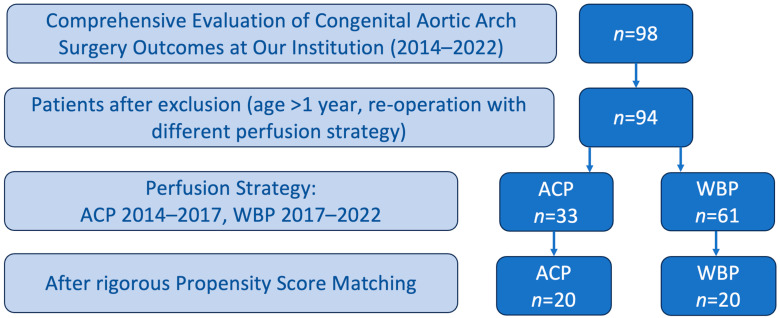
Patient selection process. ACP: Antegrade Cerebral Perfusion; WBP: Whole-Body Perfusion.

There were 98 pediatric patients who underwent aortic arch surgery at our center during this period. Four patients had to be excluded due to their age of over one year and redo surgery with another perfusion strategy. This left a cohort of 94 patients, of whom 61 patients were treated with WBP and 33 patients with ACP only. To minimize the effect of different group sizes, a strict Propensity Score Matching was conducted. This left 20 patients in each group on which the final analysis was performed. The patient selection process is displayed in Figure 1.

### 2.3. Vascular Sheath Implantation and Perfusion Strategy

**Figure 2:**
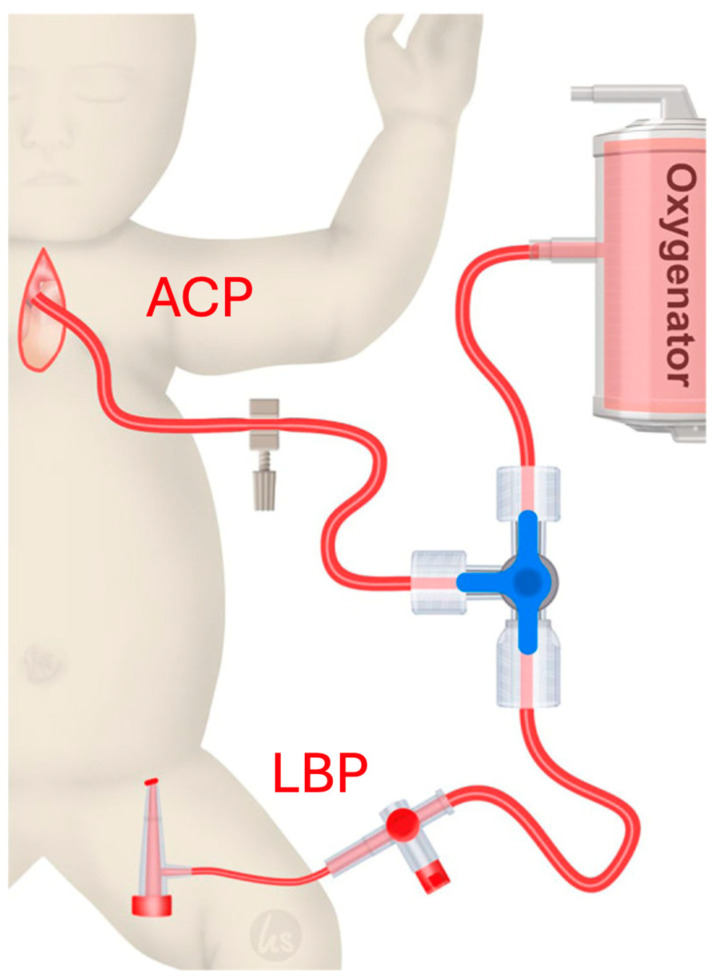
Perfusion strategy. Whole-Body Perfusion (WBP) is a combination of Antegrade Cerebral Perfusion (ACP) and Lower Body Perfusion (LBP). For ACP, the innominate artery was cannulated. LBP was achieved through an arterial sheath in the femoral artery, which was connected to an arterial line of the heart–lung machine. Patients were either treated with WBP or ACP only. Copyright by Perfusion, Sage Journals. Open Access Creative Commons Attribution-NonCommercial 4.0 License. Picture adapted from [7].

WBP is a combination of ACP and LBP (Figure 2). Implementation of LBP was performed as previously described by Sandoval Boburg et al. [7]. Briefly, an arterial sheath was inserted via ultrasound guide by the anesthesiologist in the femoral artery and was connected to an arterial line of the heart–lung machine. The size of the sheath was either 20 G, 3 or 4 French, depending on the femoral vessel size. ACP was performed in a typical way by cannulating a Polytetrafluoroethylene (PTFE) shunt previously anastomosed to the innominate artery. After cross-clamping, mild hypothermia (30–32 °C) was induced. For WBP, the femoral cannula had a flow rate of 20–40 mL/kg/min depending on the blood pressure, oxygen saturation and lactate levels for the duration of the aortic reconstruction.

For ACP, a flow rate of 50–80 mL/kg/min was used. Cerebral oxygen saturation was monitored with bifrontal cerebral Near-Infrared Spectroscopy (NIRS). Patients with ACP only were cooled down to 26–28 °C during aortic arch repair.

After completion of aortic arch repair, the neo aorta was cannulated directly, and patients were warmed up and quickly transferred to the Pediatric Intensive Care Unit (PICU). The femoral arterial sheath was then used for blood pressure monitoring and removed as soon as possible to prevent complications such as limb malperfusion or ischemia.

To mitigate risks associated with femoral arterial sheath use in neonates, such as vascular injury and limb ischemia, the protocol included continuous intraoperative monitoring of distal limb oxygen saturation. Perfusion was checked preoperatively and direct postoperatively using Doppler ultrasound and clinical assessments. Postoperatively in the PICU, patients were closely monitored for signs of vascular compromise, and early intervention was initiated in the single case of vascular complication observed.

### 2.4. Analyzed Parameters and Outcome

Demographic parameters included gender, age, height and weight. Intraoperative parameters such as maximum lactate level at reperfusion, duration of CPB, aortic cross-clamping time, lowest body temperature and intraoperative Transfusion of Red Blood Cells, Fresh Frozen Plasma or Platelets were recorded. Creatinine levels, lactate levels and hepatic parameters, including Glutamate–Oxalacetate–Transaminase (GOT), Glutamate–Pyruvate–Transaminase (GPT) and Lactate Dehydrogenase (LDH), were recorded preoperatively, upon transfer to the PICU and at 24 and 72 h postoperatively.

Regular examinations of the lower extremities were performed in the PICU at least once every 8 h to check for signs of malperfusion or limb ischemia after sheath removal. Length of Stay (LOS) in the PICU and duration of mechanical ventilation were also recorded.

The main objective was to compare major early postoperative complications between the ACP and WBP groups. Major renal complications were defined as the requirement for dialysis, following current guidelines [10]. Major neurologic complications included symptoms such as seizures and intracranial bleeding or ischemia detected on diagnostic imaging. Patients underwent transcranial ultrasound examination for major congenital malformations before surgery and twice per week post-surgery. In patients with Extracorporeal Life Support (ECLS), ultrasound was performed daily to check for any bleeding. Major gastrointestinal complications in terms of Necrotizing Enterocolitis (NEC) were defined as any postoperative impairment of the gastrointestinal system requiring treatment and prolonging the PICU stay. Multiorgan dysfunction was defined as the dysfunction of two or more organ systems based on clinical and biochemical parameters. Thirty-day mortality was recorded and compared between both groups.

### 2.5. Statistics

All statistical analyses were conducted using SPSS, Version 28.0 (IBM Corporation, Armonk, NY, USA) and Microsoft Excel (Microsoft Corporation, 2019, Redmond, WA, USA) for data organization. The normality of the data distribution was assessed using the Shapiro–Wilk test, Kolmogorov–Smirnov test and Q-Q plots. Data are reported as median and interquartile range (IQR) for non-normally distributed data and mean ± standard deviation (SD) for normally distributed data.

Propensity Score Matching (PSM) was applied to reduce selection bias inherent to retrospective studies and to achieve an optimal balance in baseline covariates between the two groups. PSM was chosen over alternative methods, such as Inverse Probability Weighting (IPW), due to its ability to provide a well-defined matched sample for direct comparison. This approach aligns with the primary goal of the study, which was to assess clinical outcomes between WBP and ACP under conditions of minimized confounding.

Although PSM resulted in the exclusion of 60% of patients, the resulting matched sample size (*n* = 20 per group) was deemed sufficient to address the study’s objectives in this single-center analysis. The chosen method ensured robust internal validity by focusing on a highly comparable subset of patients, which is critical for assessing outcomes in a rare clinical setting with limited cohort sizes.

PSM was performed using a nearest-neighbor algorithm without replacement, with a caliper width of 0.2 of the standard deviation of the logit of the propensity score. Matching variables included patient demographics (age, weight and sex), preoperative laboratory values and comorbid conditions. Standardized mean differences (SMDs) were used to evaluate balance before and after matching.

Continuous variables are presented as means and SD or median and IQR and were compared using either the Student’s *t*-test for normally distributed data or the Mann–Whitney U-test for non-normally distributed data. Ordinal variables are reported as absolute numbers and percentages and were analyzed using the Chi-square test. *p*-values ≤ 0.05 were considered statistically significant.

## 3. Results

Our retrospective analysis included 98 pediatric patients who underwent aortic arch surgery at our center. After strict propensity score matching, 20 patients remained in each group for the final analysis.

There were no significant differences between the groups regarding preoperative demographics like age, gender, height and weight. Additionally, preoperative laboratory test results showed no significant differences, as summarized in Table 1.

Table 2 presents the intraoperative results. Patients in the WBP group had significantly shorter CPB and aortic cross-clamp times (*p* < 0.001). LBP was performed with a mean duration of 35.05 ± 17.18 min (median 30.0 min, IQR (25.0–44.5)). As expected, due to different temperature management strategies implemented in each group, there was a significant difference in the lowest body temperature achieved (*p* < 0.001). Maximum lactate levels at time of reperfusion were also significantly lower in the WBP group (*p* < 0.001). Furthermore, the WBP group required significantly fewer intraoperative blood transfusions with Red Blood Cells, Fresh Frozen Plasma and Platelets (*p* ≤ 0.001).

Postoperative parameters and outcomes are shown in Table 3. Lactate levels in the ACP group remained elevated at 24 h postoperatively (*p* = 0.026) but normalized by 72 h, with no further differences between groups. The perfusion strategy did not significantly impact creatinine, GOT or LDH levels within the first 72 h after surgery. However, GPT levels were significantly lower in the WBP group immediately postoperatively (*p* = 0.003) and equalized after 24 h.

Although there was no difference in the LOS on PICU between groups, patients in the WBP group were weaned from mechanical ventilation significantly faster (*p* = 0.01) and had significantly higher urine output in the first 24 h (*p* = 0.002).

Table 4 lists the postoperative complications. While there were no statistically significant differences between the groups, there was a trend toward fewer cases of multiorgan dysfunction in the WBP group (*p* = 0.072). One patient (5%) sustained thrombotic complications due to the arterial sheath.

## 4. Discussion

Over the last few years, there have been constant attempts to reduce complications—mainly neurological or renal failure—and mortality in neonatal aortic arch surgery by refining perfusion strategies. DHCA has been associated with higher complication rates compared to ACP, making ACP a more commonly used technique. While ACP has improved outcomes, adequate visceral perfusion remains a significant challenge [2,5]. Various groups have approached this issue with different strategies. Although substantial data support WBP in adult patients, data for pediatric and neonatal patients are extremely scarce [11,12]. Most available data come from retrospective analyses of small case series, with only one prospective randomized trial identified [13].

LBP can be achieved by direct cannulation of the descending aorta, typically requiring either an additional lateral thoracotomy or direct cannulation via a posterior pericardiotomy [6,13,14,15]. While this can be a very invasive approach with the risk of injuring adjacent structures, it also narrows the already small operating field. Also, at some point, LBP can not be maintained for the whole time needed to complete the aortic anastomosis.

A newer and less common approach achieved LBP through cannulation of the femoral artery [1]. This technique was first described by Rajagopal et al. in 2010. In our institution, we adapted from ACP to WBP with this technique in 2017, which has become our standard since then. To highlight some benefits of this technique, it does not interfere with the operating field, the arterial sheath can be applied prior to surgery without prolonging operating time, and it is a less invasive and safer method than direct aortic cannulation. Over the years, we have managed to perform all of these challenging surgeries with the same experienced team of surgeons, anesthesiologists, and perfusionist. This maintained a high standard and consistency in our outcome. To our knowledge, we are among the few centers with a relatively large cohort of patients treated using this method. Yamamoto et al. described the feasibility of this technique from their clinical experience but did not provide any data on patient outcomes [16,17]. Recently, a group from Berlin conducted a similar comparison of WBP versus ACP, with a cohort size comparable to ours. Their key findings align with our results: WBP was associated with reduced kidney injury, fewer intraoperative transfusions and lower lactate levels at the time of reperfusion. However, they achieved WBP through either direct cannulation of the descending aorta or a femoral arterial sheath. While their study clearly shows the benefits of WBP, it does not differentiate which technique was used for WBP. Therefore, the consistency of the procedure must be appraised critically [18].

The primary risks associated with femoral arterial sheath use are punction-related complications and limb malperfusion or ischemia. In our Propensity Score-matched cohort, only one patient (5%) experienced a vascular complication of this nature, and in the overall cohort, the incidence was 4 out of 61 patients (6.6%).

In our study, CPB and aortic cross-clamp times were significantly shorter in the WBP group—approximately half the time of the ACP group. Additionally, patients in the ACP group had a lower minimal body temperature. The shorter CPB time can be attributed to less extensive cooling and re-warming. However, the shorter cross-clamp time remains unexplained. The reduced duration of extracorporeal circulation, higher body temperature, and consequently, lower incidence of capillary leak syndrome and coagulopathy likely contributed to the significantly reduced need for intraoperative transfusions of Red Blood Cells, Fresh Frozen Plasma and Platelets in the WBP group.

Although no significant differences in postoperative creatinine levels or dialysis rates were observed between the groups, WBP-treated patients exhibited higher urine output in the first 24 h, possibly indicating a fewer occurrence of kidney injury in the WBP group. Notably, GPT levels were significantly lower in the WBP group after 24 h, whereas GOT levels showed no significant difference. Given that GPT is liver-specific and a sensitive marker for liver damage, particularly in ischemic hepatitis, this finding suggests better hepatic outcomes with WBP [19]. Lactate, indicative of hypoxia and malperfusion, was elevated at the time of reperfusion and after 24 h in the ACP group. Interestingly, after 72 h, there were no changes in laboratory parameters and urine output detectable anymore, with both groups showing a return to baseline levels. While WBP-treated patients had a significantly shorter duration of ventilator dependence, no difference was observed in the length of PICU stay between the groups.

Regarding major postoperative complications like dialysis, neurological or bleeding complications, NEC, Thorax apertum or ECLS, no differences were detectable between groups. Nevertheless, there was a trend towards less multiorgan dysfunction in the WBP group.

Our findings align with prior studies that highlight the benefits of WBP in improving intraoperative and early postoperative outcomes. Previous research has shown that maintaining LBP during neonatal aortic arch surgery reduces the risk of organ dysfunction, particularly renal and hepatic impairment. This study adds to the growing body of evidence supporting the feasibility of WBP, especially via a femoral artery sheath, particularly in centers with appropriate expertise and monitoring protocols.

We hope that our study contributes to the rare existing data in this field. This may encourage other groups to improve their standards to obtain better outcomes for neonates and infants in need of aortic arch surgery.

## 5. Limitations

This study has several limitations. The retrospective design inherently carries the risk of selection bias and limits the ability to establish causality. Additionally, the short follow-up period restricts our ability to assess long-term outcomes and potential late complications. The relatively small sample size, which is the consequence of being a single-center study, further limits the generalizability of our findings. A multicenter study with a larger patient cohort and extended follow-up would provide more robust data and potentially validate our results.

Although we managed to perform all of these surgeries with the same team of surgeons, anesthesiologists and perfusionist over the years, which provides high consistency, a bias due to growing personal experience and changes over time cannot be excluded.

One limitation of the study is the reduction in sample size due to PSM, which excluded approximately 60% of patients. While alternative methods like IPW could have preserved the entire cohort, we prioritized achieving a well-matched, unbiased sample to ensure valid comparisons between the groups. This decision was guided by the study’s primary goal of assessing clinical outcomes under conditions of minimized confounding. Future studies with larger, multicenter cohorts could explore the use of IPW to address this limitation.

Another limitation of this study is the focus on immediate postoperative outcomes without an assessment of long-term effects, such as neurodevelopmental status, renal function and survival rates. While our results demonstrate the potential benefits of WBP during the perioperative period, they do not provide insights into whether these benefits translate into sustained improvements over time. Given the importance of long-term outcomes, particularly in neonatal populations, future studies should include prospective follow-up to evaluate neurodevelopmental progress, renal function and overall survival. Such data would provide a more comprehensive understanding of the lasting impact of WBP compared to ACP.

The use of a femoral arterial sheath in neonates carries inherent risks, including vascular injury and limb ischemia. Although our monitoring protocols successfully identified and managed the single vascular complication, the absence of long-term limb outcome data remains a limitation. Future studies should include follow-up assessments of limb function and vascular integrity to ensure a comprehensive evaluation of this technique.

Further investigations should aim to address these limitations to enhance the reliability and applicability of the findings.

## 6. Conclusions

Our findings underscore the significant advantages of using LBP via a femoral artery sheath as a method for WBP in neonatal aortic arch surgery. This method not only simplifies the surgical process but also markedly improves clinical outcomes. Patients undergoing WBP required fewer intraoperative blood transfusions, experienced significantly shorter CPB and aortic cross-clamp times, and demonstrated enhanced postoperative recovery, including quicker weaning from mechanical ventilation. Moreover, the trend towards reduced multiorgan dysfunction highlights the potential for this approach to minimize serious postoperative complications. These results suggest that femoral artery-based WBP could represent a paradigm shift in neonatal aortic arch surgery, offering a safer and more efficient strategy. However, larger multicenter studies are essential to validate these promising outcomes and explore their impact on patient health.

## Figures and Tables

**Table 1:** Preoperative demographic and laboratory parameters.

	Total Cohort (*n* = 40)	WBP (*n* = 20)	ACP (*n* = 20)	*p*-Value
*DEMOGRAPHICS*, mean (±SD) or median (IQR)				
Age [d]	12.0 (8.0–22.0)	12 (8–22)	11 (9–21)	0.684 ^MW^
Gender	25	11	14	0.327 ^Chi2^
Height [cm]	50.0 (49.0–53.0)	50 (49–53)	51 (50–54)	0.333 ^MW^
Weight [kg]	3.68 ± 1.32	3.81 ± 1.79	3.55 ± 0.58	0.744 ^T-T^
*PREOPERATIVE PARAMETERS*, mean (±SD) or median (IQR)				
Creatinine [mg/dL]	0.50 ± 0.23	0.52 ± 0.24	0.49 ± 0.23	0.688 ^T-T^
GOT [U/L]	31.5 (27.0–46.25)	31.5 (27–46.25)	30 (28–44)	0.531 ^MW^
GPT [U/L]	16.0 (9.25–28.0)	16 (9.25–28)	15 (8–26)	0.606 ^MW^
LDH [U/L]	318.5 (267.5–454.0)	454 (267.5–471)	318.5 (267.5–349)	0.099 ^MW^

GOT: Glutamate–Oxalacetate–Transaminase; GPT: Glutamate–Pyruvate–Transaminase; LDH: Lactate Dehydrogenase; MW: Mann–Whitney U-test; Chi^2^: Chi-square test; T-T: Student’s *t*-test.

**Table 2:** Intraoperative parameters.

	Total Cohort (*n* = 40)	WBP (*n* = 20)	ACP (*n* = 20)	*p*-Value
*SURGICAL PARAMETERS*, mean (±SD) or median (IQR)				
CPB Time [min]	129.80 ± 63.14	86.65 ± 25.47	172.95 ± 60.12	**<0.001 ^T-T^**
Cross-Clamp Time [min]	61.5 (43.5–87.75)	48.5 (34.0–60.0)	85.5 (65.25–94.0)	**<0.001 ^MW^**
LBP Time [min]		30.0 (25.0–44.5)		
Min. Body Temperature [°C]	29.95 (26.775–32.75)	32.0 (30.65–34.75)	26.85 (25.8–28.0)	**<0.001 ^MW^**
Max. Lactate at Reperfusion [mmol/L]	2.05 (1.625–4.175)	1.7 (1.325–1.975)	4.15 (3.475–5.4)	**<0.001 ^MW^**
*INTRAOPERATIVE TRANSFUSION*, median (IQR)				
Red Blood Cells [mL]	300.0 (177.5–565.0)	245.0 (110.0–315.0)	530.0 (300.0–775.0)	**<0.001 ^MW^**
Fresh Frozen Plasma [mL]	300.0 (162.5–387.5)	200.0 (100.0–292.5)	375.0 (300.0–600.0)	**<0.001 ^MW^**
Platelets [mL]	60.0 (32.5–300.0)	60.0 (26.25–60.0)	300.0 (87.5–337.5)	**0.001 ^MW^**

CPB: Cardio-Pulmonary Bypass; LBP: Lower Body Perfusion; MW: Mann–Whitney U-test; T-T: Student’s *t*-test. Bold in the *p*-value column marks statistically significant values.

**Table 3:** Postoperative parameters and outcome.

	Total Cohort (n = 40)	WBP (n = 20)	ACP (n = 20)	*p*-Value
*POSTOPERATIVE PARAMETERS,* median (IQR)				
Lactate [mmol/L]				
24 h	1.55 (1.125–2.300)	1.1 (0.7–1.6)	2.3 (1.55–2.3)	**0.026 ^MW^**
72 h	1.20 (0.90–1.58)	1.2 (0.9–1.6)	1.2 (0.9–1.6)	0.635 ^MW^
Creatinine [mg/dL]				
24 h	0.60 (0.40–0.70)	0.5 (0.4–0.6)	0.6 (0.4–0.7)	0.651 ^MW^
72 h	0.50 (0.40–0.60)	0.5 (0.4–0.6)	0.6 (0.4–0.6)	0.122 ^MW^
GOT [U/L]				
postop	81.0 (51.50–102.50)	68.0 (48.0–95.50)	84.0 (61.25–129.75)	0.107 ^MW^
24 h	47.0 (34.0–66.5)	42.50 (34.0–53.75)	49.0 (33.25–88.50)	0.239 ^MW^
72 h	25.0 (17.25–42.0)	28.0 (20.25–41.0)	21.50 (17.00–42.75)	0.379 ^MW^
GPT [U/L]				
postop	16.0 (10.25–21.75)	12.50 (8.00–18.25)	20.0 (16.00–28.75)	**0.003 ^MW^**
24 h	11.0 (8.0–19.0)	9.00 (6.25–18.25	12.50 (9.25–26.50)	0.143 ^MW^
72 h	8.0 (4.0–14.75)	7.50 (4.25–14.25)	8.50 (4.00–14.25)	0.946 ^MW^
LDH [U/L]				
postop	438.0 (327.25–548.75)	360.0 (279.0–491.5)	438.0 (327.25 548.75–491.5)	0.976 ^MW^
24 h	360.0 (279.0–491.5)	323.5 (283.75–458.50)	371.5 (279.0–521.25)	0.482 ^MW^
72 h	323.5 (264.25–444.25)	338.50 (260.75–478.50)	312.0 (264.25–417.50)	0.839 ^MW^
*POSTOPERATIVE OUTCOME*				
LOS on Ventilator [d]	6.0 (3.0–9.5)	6.0 (3.0–9.5)	9.5 (6.0–12.5)	**0.010 ^MW^**
LOS at PICU [d]	11.0 (8.0–16.5)	11.0 (8.0–16.5)	14.0 (10.0–18.0)	0.489 ^MW^
Urine Output [mL]				
24 h	127.5 (82.0–213.5)	213.5 (127.5–320.5)	87.0 (71.25–140.50)	**0.002 ^MW^**
72 h	566.5 (431.5–667.25)	667.3 (566.5–800.0)	504.50 (364.0–688.25)	0.291 ^MW^

GOT: Glutamate-Oxalacetate-Transaminase; GPT: Glutamate-Pyruvate-Transaminase; LDH: Lactate Dehydrogenase; LOS: Length of Stay; PICU: Pediatric Intensive Care Unit; MW: Mann-Whitney U-test. Bold in the *p*-value column marks statistically significant values.

**Table 4:** Postoperative complications.

	Total Cohort (*n* = 40)	WBP (*n* = 20)	ACP (*n* = 20)	*p*-Value
*POSTOPERATIVE COMPLICATIONS,* n (%)				
Dialysis		0 (0%)	1 (5%)	0.311 ^Chi2^
Neurological Deficits		1 (5%)	0 (0%)	0.311 ^Chi2^
30-day Mortality		1 (5%)	0 (0%)	0.311 ^Chi2^
Thorax apertum		8 (40%)	12 (60%)	0.206 ^Chi2^
NEC		0 (0%)	2 (10%)	0.147 ^Chi2^
Multiorgan Dysfunction		0 (0%)	3 (15%)	0.072 ^Chi2^
Bleeding Complications		1 (5%)	2 (10%)	0.548 ^Chi2^
Vascular complications due to arterial sheath		1 (5%)		
ECLS intraop		2 (10%)	4 (20%)	0.376 ^Chi2^
ECLS postop		2 (10%)	1 (5%)	0.548 ^Chi2^

NEC: Necrotizing Enterocolitis; ECLS: Extracorporeal Life Support; Chi^2^: Chi-square test.

## Data Availability

The data underlying this article are available in the article.
